# An Apriori Algorithm-Based Association Analysis of Analgesic Drugs in Chinese Medicine Prescriptions Recorded From Patients With Rheumatoid Arthritis Pain

**DOI:** 10.3389/fpain.2022.937259

**Published:** 2022-07-25

**Authors:** Wei-dong Lai, Dian-ming Li, Jie Yu, Lin Huang, Ming-zhi Zheng, Yue-peng Jiang, Song Wang, Jun-jun Wen, Si-jia Chen, Cheng-ping Wen, Yan Jin

**Affiliations:** ^1^The Second Clinical Medical College of Zhejiang Chinese Medical University, Hangzhou, China; ^2^College of Basic Medical Science, Zhejiang Chinese Medical University, Hangzhou, China; ^3^Hangzhou AI Center, China Academy of Information and Communications Technology, Hangzhou, China

**Keywords:** rheumatoid arthritis (RA), chronic pain, data mining, Apriori Algorithm, analgesic drugs

## Abstract

Chronic pain, a common symptom of people with rheumatoid arthritis, usually behaves as persistent polyarthralgia pain and causes serious damage to patients' physical and mental health. Opioid analgesics can lead to a series of side effects like drug tolerance and addiction. Thus, seeking an alternative therapy and screening out the corresponding analgesic drugs is the key to solving the current dilemma. Traditional Chinese Medicine (TCM) therapy has been recognized internationally for its unique guiding theory and definite curative effect. In this study, we used the Apriori Algorithm to screen out potential analgesics from 311 cases that were treated with compounded medication prescription and collected from “Second Affiliated Hospital of Zhejiang Chinese Medical University” in Hangzhou, China. Data on 18 kinds of clinical symptoms and 16 kinds of Chinese herbs were extracted based on this data mining. We also found 17 association rules and screened out four potential analgesic drugs—“Jinyinhua,” “Wugong,” “Yiyiren,” and “Qingfengteng,” which were promised to help in the clinical treatment. Besides, combined with System Cluster Analysis, we provided several different herbal combinations for clinical references.

## Introduction

Rheumatoid Arthritis (RA), a chronic, systemic inflammatory disease, is often characterized by synovitis, symmetry, and multiple arthritis, and especially occurs on the limb facet joints (1–4). In addition to joint deformities that occur in the local area, dysfunction of other organs is also involved (5–7). For example, RA patients also showed interstitial lung disease (8, 9), acute coronary syndrome (10–12), and other serious systemic lesions (13–19). However, during RA, the stubborn symptom—“pain”—often runs through pathological changes, while among all pain types, chronic inflammatory pain dominates (20–27). Therefore, treatments of arthritis pain in RA patients usually focus on the way of controlling inflammation (28, 29). As for the refractory rheumatoid arthritis treatment, “Immunosuppressants” like “Methotrexate” can effectively control the inflammatory pain of RA in its active period, but show a poor therapeutic effect on the chronic pain (30). After the usage of nonsteroidal anti-inflammatory drugs, approximately 20% of RA patients still show subjective symptoms of chronic pain (31). Although Disease-modifying anti-rheumatic drugs (DMARDs) and other drugs can alleviate the arthritis pain of RA through antiinflammation to a large extent, patients have difficulty accepting long-term medication with their side effects (30, 32, 33). In addition, the usage of opioids for pain relief has addictive and resistant side effects (34–37). Thus, complementary and alternative therapies for additional remission and fewer side effects are still needed.

Nowadays, the acceptance of Traditional Chinese Medicine (TCM) in clinics has been increasing around the world (38). “Aconiti Radix Cocta” (“AC”), a kind of herb used in “Expelling wind,” “Removing dampness,” and “Relieving pain,” is found to have effects in reducing paw swelling, attenuating inflammation and bone destruction in joint tissues, and reducing IL-1β and IL-17A in the serum of collagen-induced arthritis (CIA) rats (39). Moreover, according to a randomized controlled trial (RCT) study associated with the curative effect of TCM on RA in 2007, the patients in the TCM group with “Ganoderma lucidum” (“Ling Zhi”) and “San Miao San” (“SMS”) had received a robust analgesic effect. Compared with placebo, pain sensitivity had been improved in the TCM group and the percentage of IL-18 released was lower in the *ex-vivo* experiment test (40). In addition to a single herb, the compounded medication prescription like “Guizhi-Shaoyao-Zhimu decoction” can also receive positive curative effects. Recent clinical research has shown that the clinical cure rate of “Guizhi-Shaoyao-Zhimu decoction” for RA patients is around 90%, which is higher than that of indomethacin, tripterygium glycosides, and prednisonec (41). It infers that TCM is a proper alternative option in the treatment of rheumatoid arthritis pain.

In TCM theory, RA is believed to be caused by “attacks of wind, cold, damp humor” and the herbs with beneficial effects of expelling wind and removing dampness were prescribed to relieve pain (39). In addition, the concepts of “Four Properties” (“Cool,” “Warm,” “Hot,” and “Cold”) and “Five Tastes/flavors” (“Sour,” “Sweet,” “Bitter,” “Pungent,” and “Salty”) are used to interpret and describe the pathological changes of diseases and the classification of natural herbs, and different combinations of the “four properties” and “five tastes/flavors” lead to the various effects of the herbs (42). Apart from Yin and Yang categories, TCM natural herbs are also classified according to their “cool” and “hot” properties. For example, “Cool” herbs usually have bitter, salty, and sour tastes, but “hot” ones usually have pungent and sweet tastes (43). Furthermore, in TCM theory, herbs with “cool” or “cold” properties are used to clear “hot” and may show functions of tonifying “Qi”-deficiency, relieving the patient's severe symptoms like pain induced by inflammation. On the contrary, herbs with “warm” or “hot” properties like “Fuzi” are always regarded as “interior-warming medicine” and could be used to resist the inside or outside “cold” (44–46).

Apriori algorithm is one of the classic algorithms for data mining. Compared with other algorithms, the iterative method of searching layer by layer in the horizontal organization shows the advantages of the “Apriori Algorithm” in dealing with data of smaller frequent itemsets. Correspondingly, numerous studies related to TCM data mining adopted the “Apriori Algorithm” to analyze the drug compatibility and summarize the treatment experience of the clinical practitioners (47–50). Here, we chose the Apriori algorithm to screen out the traditional Chinese medicine prescription with potential analgesic effects, from 311 clinical cases with chronic pain in rheumatoid arthritis. All compounded medication prescriptions were collected from “Second Affiliated Hospital of Zhejiang Chinese Medical University” and were input into the database of “Rheumatism Intelligent Auxiliary diagnosis and treatment system” to deeply explore and analyze the prescribing patterns in the treatment of RA pain. The purpose of this study is to provide a new idea for selecting complementary and alternative therapies in the clinic, as well as potential analgesics, for patients with chronic pain in rheumatoid arthritis.

## Materials and Methods

### Data Sources

All 311 cases of effective prescriptions for chronic pain in rheumatoid arthritis come from the “Second Affiliated Hospital of Zhejiang Chinese Medical University.” The relevant data like symptoms of chronic pain in patients with rheumatoid arthritis and the following drugs prescribed by doctors have been input into the database of “Rheumatism Intelligent Auxiliary Diagnosis and Treatment System” (http://106.12.195.182/).

All patients with RA pain symptoms in the database of “Rheumatism Intelligent Auxiliary Diagnosis and Treatment System” were selected as the research objects, and their corresponding pulse case prescriptions were selected as the sources of symptoms and traditional Chinese medicine data. All tasks involving data input, data maintenance, and data output were carried out by third-party researchers who are skilled in data research and are in charge. Two independent researchers are responsible for data input, verification, and analysis, respectively.

### Inclusion Criteria

1) Meet the diagnostic criteria of rheumatoid arthritis (2010 ACR/EULAR diagnostic criteria).
2) The patients present with symptoms of chronic pain. (The duration of the disease ≥6 months).
3) Patients' age ranged from 18 to 70 years.
4) 5.1 > DAS28 > 3.2 (Before treatment) and DAS28 < 2.6 (After treatment).
5) Health Assessment Questionnaire-Disability Index (HAQ-DI) ≥1 (Before treatment) and HAQ-DI <0.5 (After treatment).
6) Patients' VAS scores had decreased after taking the TCM treatment.
7) Each of the effective cases is provided from “The Second Affiliated Hospital of Zhejiang Chinese Medical University” and the medical record information is complete and there are no data missing among the prescriptions.
8) Prescription physician (deputy) director of Chinese medicine practitioners.
9) Patient signed an informed consent form.

### Exclusion Criteria

1) Does not meet the diagnostic criteria for rheumatoid arthritis (2010 ACR/EULAR).
2) The chronic pain symptoms does not exist.
3) Patients' VAS scores and DAS28 scores did not decrease after treatment.
4) Liver function consisting of ALT and AST is greater than or equal to 2 times the upper limit screening.
5) White Blood Cells in Blood routine count is equal to or less than 3.5^*^10^9^/L.
6) Patients with serious basic diseases and poor control, or have serious complications.
7) Allergic drugs were involved in the patient's prescription.
8) Only accept pure western medicine treatment without traditional Chinese medicine treatment.

### Standardization of Terminology

#### Standardization of Traditional Chinese Medicine Names

To standardize the description of drug names, all traditional Chinese medicine names in this study are the same as the name used in the 2020 edition of the Pharmacopeia of the People's Republic of China (2020 edition). In principle, the words with regional characteristics in the drug names are rejected, such as “Han Fangji” named “Fangji,” “Panax notoginseng powder” named “Sanqi,” and so on. It is worth noting that “Chuanniuxi” and “Niuxigen” are collectively referred to as “Huainiuxi” in the Pharmacopeia of the People's Republic of China (2020 edition), but given the existence of two drugs appearing in the same prescription at the same time, the names of such drugs are not changed. Besides, the names of two herbs will not be renamed if they belong to different parts of the same plant, such as “Jinyinhua” and “Rendongteng.”

#### Standardization of Clinical Symptom Names

To unify the clinical symptoms with the same meaning and reduce the presence of symptoms associated with excessive low frequency, some pain-related symptoms are combined according to the location of pain, “Pain in interphalangeal joints,” “Wrist pain,” “Elbow pain,” and “Shoulder pain” are described as “upper limb joint pain,” “Ankle pain” and “Knee joint pain” are described as “Lower extremity joint pain,” “Lumbar pain,” “Hip pain,” and “Sacral joint pain” are described as “Lumbosacral pain.” The pain enrolled in two or more parts of the body is described as “multi-joint pain”; The symptoms of “Pain improvement” and “Pain relief” are described as “Pain reduction,” “Difficulty bending” and other symptoms were replaced as “Unfavorable activity,” “Limb soreness” and “Neck discomfort” were generalized as “Limb discomfort,” “Fatigue” and “Burnout” were unified as “Fatigue.”

### Case Screening and Data Audit

**Figure 1:**
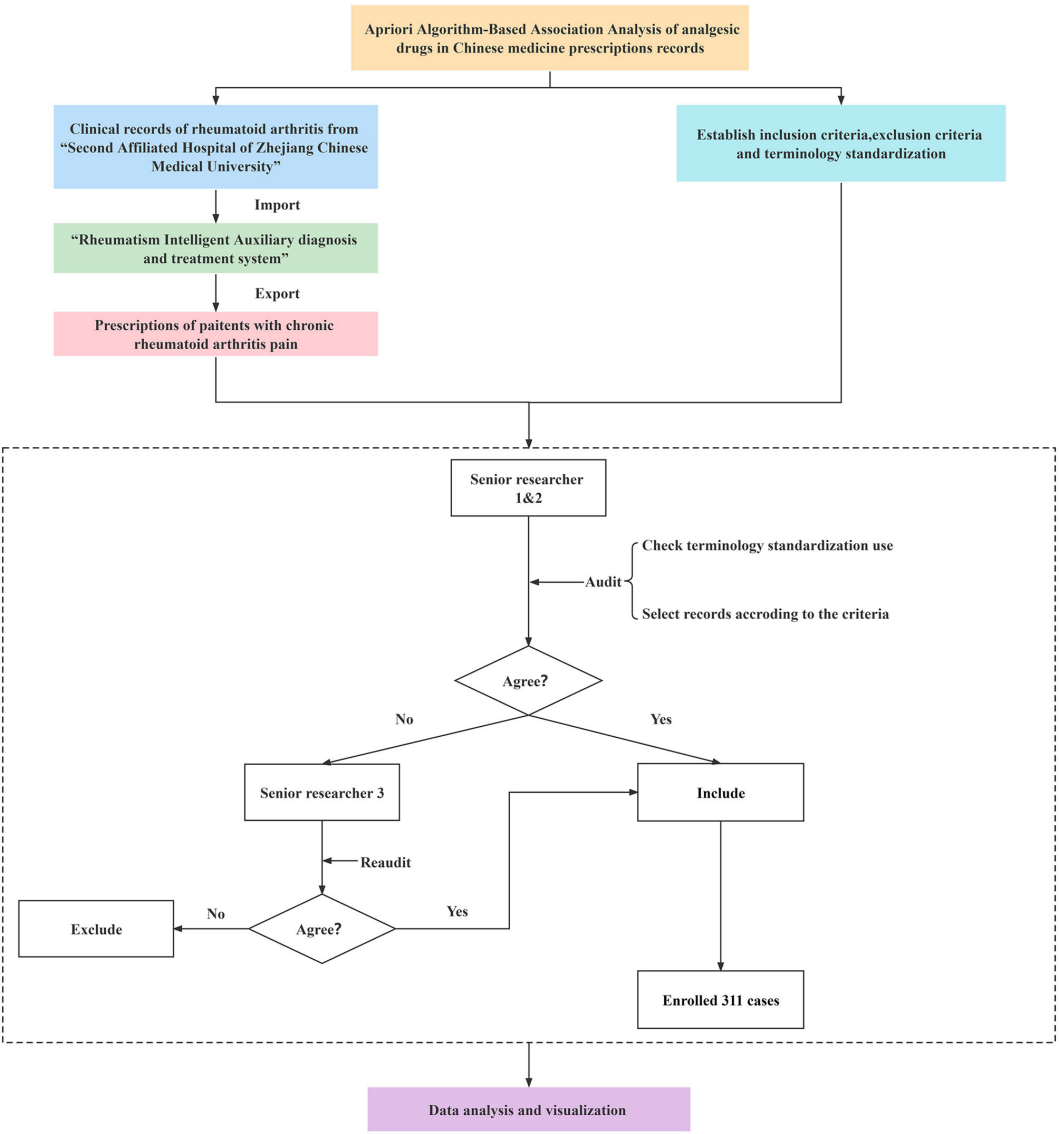
Flow diagram of Apriori Algorithm-Based Association Analysis of analgesic drugs.

“Rheumatism Intelligent Assisted Diagnosis and Treatment System” is an independent research and development database platform invented by “the Institute of Rheumatology Immunology, Zhejiang University of Traditional Chinese Medicine” and collecting prescriptions from “The Second Affiliated Hospital of Zhejiang Chinese Medical University.” Based on the “Rheumatism Intelligent Assisted Diagnosis and Treatment System” database, all cases of rheumatoid arthritis including symptoms of “Chronic pain” were identified by two independent researchers, respectively. In the process of data audit, two senior researchers were asked to carry out the data audit, verify the cases whether they were meeting the criteria, and check the standardization of terminology. While the opinions of the two researchers are inconsistent, the third researcher would be asked to recheck (Figure 1).

### Data Analysis

#### Proportion Analysis

After exporting the cases from the “Rheumatism Intelligent Auxiliary Diagnosis and Treatment System,” all corresponding symptoms and prescription drugs of traditional Chinese medicine in the medical records were standardized. We counted the number of occurrences each symptom appeared in 311 prescriptions. “Graph Pad Prism 8 Software” and “Adobe Illustrator CS5” were used to express the proportions of “pain” and “other” symptoms.

#### Frequency Analysis

In this study, the frequency analysis can be divided into two categories: high-frequency drug frequency analysis and clinical symptom frequency analysis. According to the value of their frequency, the herbs and symptoms were sorted in descending order, respectively. We selected both symptoms and herbs with a frequency higher than 10%. Each corresponding data were inputted into “Graph Pad Prism 8 Software.” Finally, histograms were drawn by “Graph Pad Prism 8 Software.”

#### Classification of High-Frequency Drugs

Combined with the Pharmacopeia of the People's Republic of China (2020 edition), all high-frequency herbs were divided into different categories according to their efficacy. The number of each corresponding type was counted and the proportion of corresponding types was calculated. “Microsoft Office EXCEL2019” was chosen to draw the ring diagram to make high-frequency drug classification.

#### Network Mapping

Generally, network mapping shows the co-relationship among several objects. In this study, the co-relationships between each herb and its corresponding “Four properties,” “Five flavors,” and “Channel Tropism” were expressed by network mapping. When there was a connection between every two objects, correspondingly, they would be indicated by the “direct” in the “Microsoft Office EXCEL2019,” The data were imported into the “Cytoscape” (3.7.1 version) to draw the network map.

#### Apriori Algorithm-Based Association Rule Analysis

In this study, the second- and third-order Apriori association rules in association analysis were used to analyze the bilateral herb associations and the correlation between herbs and symptoms *via* “K-means clustering” (Association rules with frequencies less than “1” are eliminated). The preceding (LHS) and following (RHS) terms are two different objects used for association comparison, while “Confidence,” “Support,” and “Lift” are different expressions of the degree of correlation between the preceding and the following items of data. “Confidence” is the ratio of the items containing both the LHS and RHS to the items containing the former. “Support” represents the proportion of all transactions that include both the LHS and the RHS, while “Lift” is the ratio of “the proportion of things that include LHS” to “the proportion of things that include RHS.”

The values of “LHS,” “RHS,” “Confidence,” “Support,” and “Lift” were obtained by “K-mean clustering.” Then, the data were imported into “Python” (3.8.0 version). Finally, the bubble diagrams were obtained by using the installation package “Mat plot” of “Python.”

The “Lift” reflects the correlation between LHS and RHS in the association rules. The higher the data is (data >1), the higher the positive correlation it refers to. Similarly, the lower the data is (data <1), the higher the negative correlation it hints; if the degree of promotion equals or lower than “1” that means the two items shouldn't be included to analysis. To reduce the error, generally, we use the “lift” to indicate the degree of correlation between LHS and RHS.

In this study, the association rule with its value of “Confidence,” “Support,” and “Lift” higher than “50,” “10,” and “1.2,” respectively, was accepted.

#### System Cluster Analysis

The corresponding symptoms and high-frequency drug names were filled in the “Office 2019-EXCEL” by sorting out 311 RA cases, respectively. If there is a correlation between the symptoms and the corresponding drug, the relation between the two should be denoted as “1,” or it would be noted as “0.” Herbs with their cumulative number of occurrences in prescriptions higher than “50” were included in this cluster analysis. The tree diagram is made by using the system clustering analysis *via* the “hclust” installation package of the “R software” (4.1.2 version).

### Ethical Audit

This study had been reviewed and approved by the relevant ethics committee of the hospital and each patient enrolled in this study had signed an informed consent form (Ethics Approval NO: 2019-037; Version of Ethics Approval: 2.0).

## Results

### Multi-Articular Pain in the Extremities Is a Major Symptom in the 311 Cases Collected With Chronic Pain

**Figure 2:**
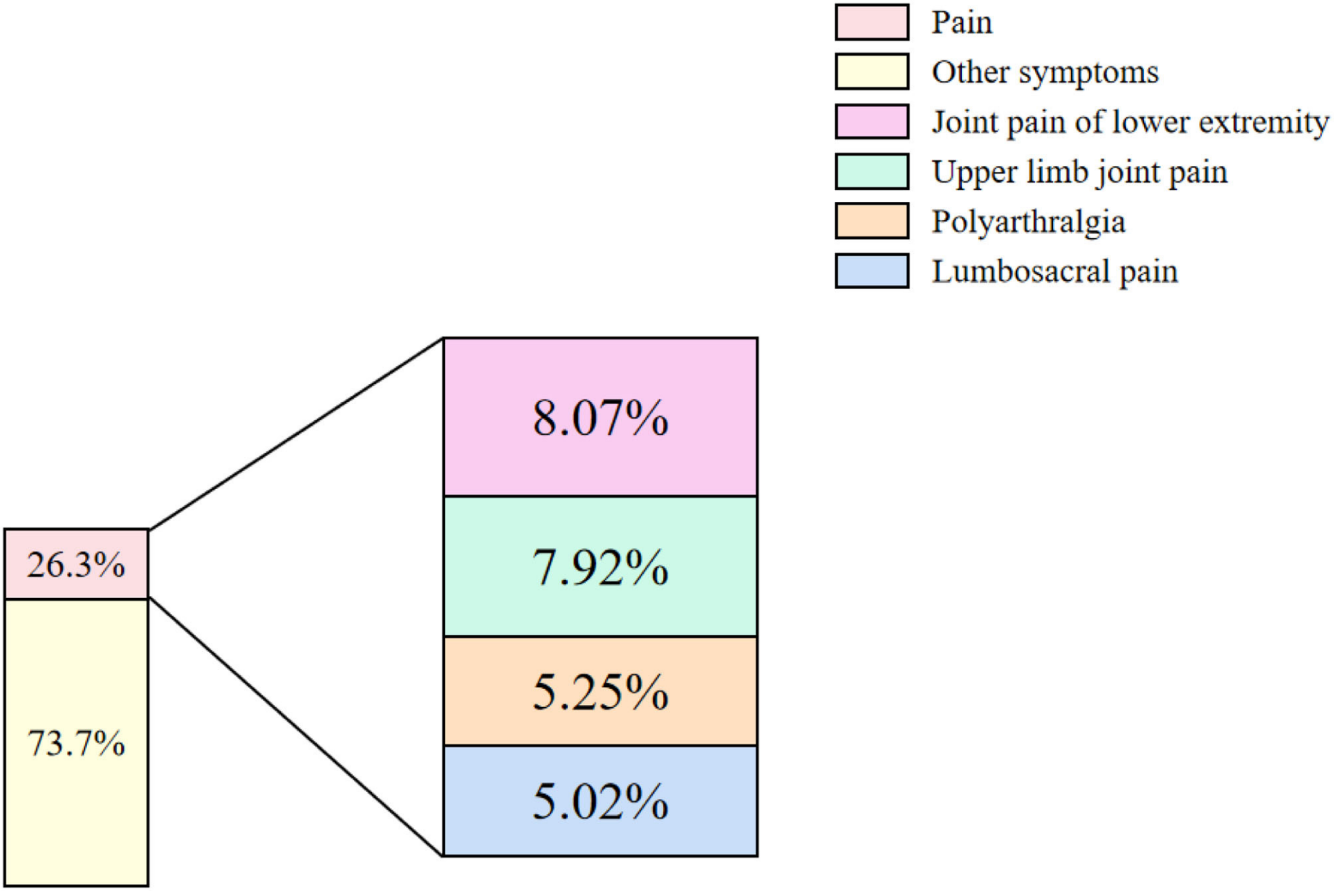
Proportions of different pain symptoms. The proportions of symptoms from 311 cases were classified into two kinds: “pain” and “other symptoms” (On the left). The pain symptoms mainly consisted of four kinds: “Joint pain of lower extremity,” “Upper limb joint pain,” “Polyarthralgia,” and “Lumbosacral pain” (On the right). Pink: Pain; Pastel yellow: Other symptoms; Purple: Joint pain of lower extremity; Green: Upper limb joint pain; Earthy yellow: Polyarthralgia; Blue: Lumbosacral pain.

Chronic pain in rheumatoid arthritis, along with other high-frequency symptoms, often endangers the physical and mental health of patients and is the main complaint of these 311 cases collected. Identifying the proportion of high-frequency symptoms is beneficial to symptomatic treatment in a clinic. Thus, we counted the clinical symptoms in the records and all symptoms of patients were collected and divided into“pain” and “other” symptoms. We found that pain symptoms accounted for 26.3%, while other symptoms accounted for 73.7%. Then, we analyzed the location of pain symptoms in the body in patients. It showed that pain was mainly located in “Joint pain of lower extremity” (8.07%), “Upper limb joint pain” (7.92%), “Polyarthralgia” (5.25%), and “Lumbosacral pain” (5.02%), respectively (Figure 2).

**Figure 3:**
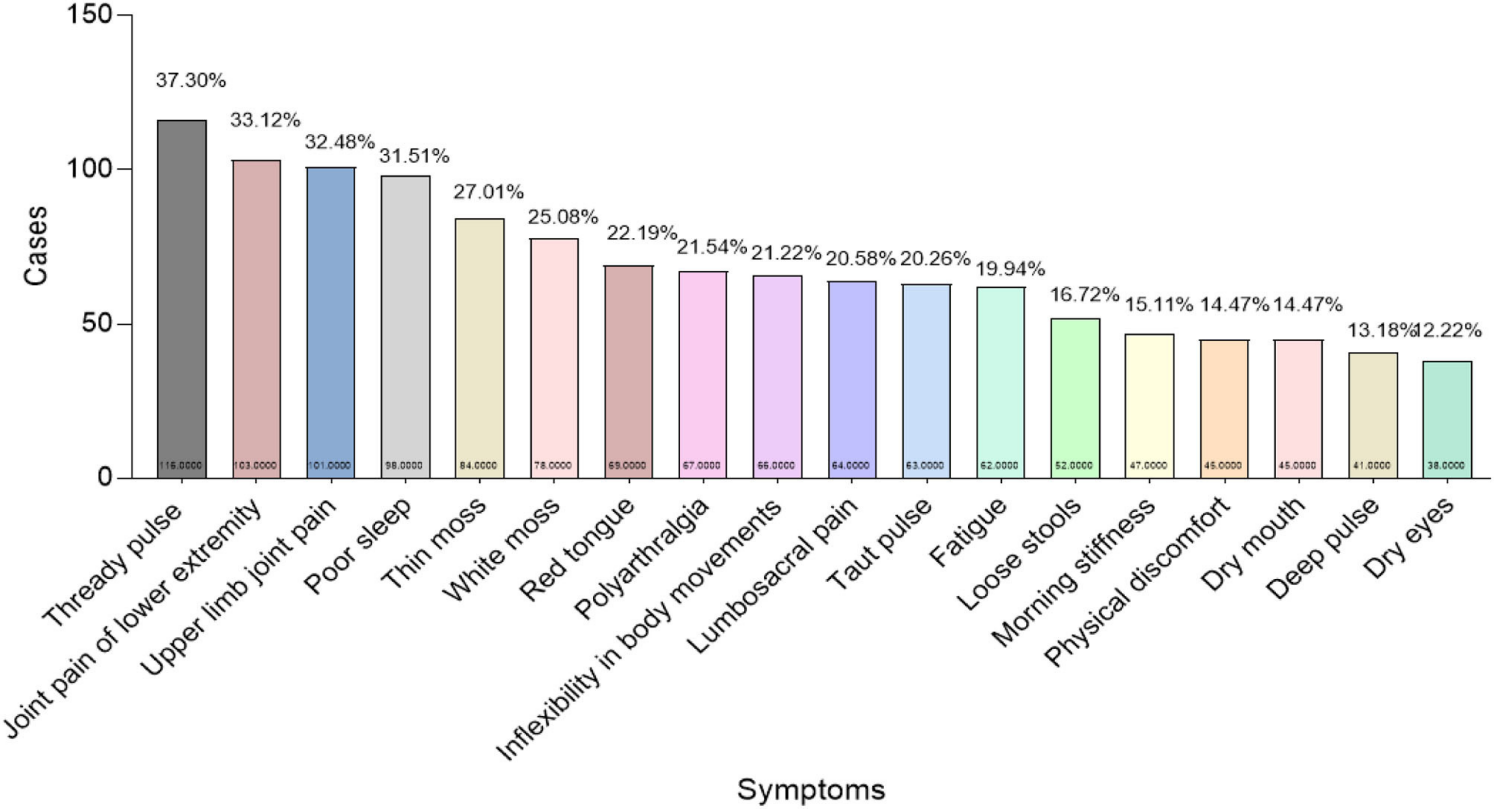
Frequency of clinical symptoms of patients with chronic pain from rheumatoid arthritis. *X*-axis: Clinical symptoms; *Y*-axis: Total cases as well as the frequency that each symptom appeared in 311 cases (At the bottom and top of the column, respectively). Symptoms with their frequencies higher than 10% were enrolled in this study.

Since a single patient can present with several symptoms, we counted and analyzed the patient cases with the same symptoms. We found 18 kinds of high-frequency symptoms that chronic RA patients behaved (the frequency higher than 10%): “Thready pulse” (116, 37.3%), “Joint pain of lower extremity” (103, 33.12%), “Upper limb joint pain” (101, 32.48%), “Poor sleep” (98, 31.51%), “Thin moss” (84, 27.01%), “White moss” (78, 25.08%), “Red tongue” (69, 22.19%), “Polyarthralgia” (67, 21.54%), “Inflexibility in body movements” (66, 21.22%), “Lumbosacral pain” (64, 20.58%), “Taut pulse” (63, 20.26%), “Fatigue” (62, 19.94%), “Loose stools” (52, 16.72%), “Morning stiffness” (47, 15.11%), “Physical discomfort” (45, 14.47%), “Dry mouth” (45, 14.47%), “Deep pulse” (41, 13.18%), and “Dry eyes” (38, 12.22%; Figure 3).

### High Frequency Used Herbs in the 311 Prescriptions

**Figure 4:**
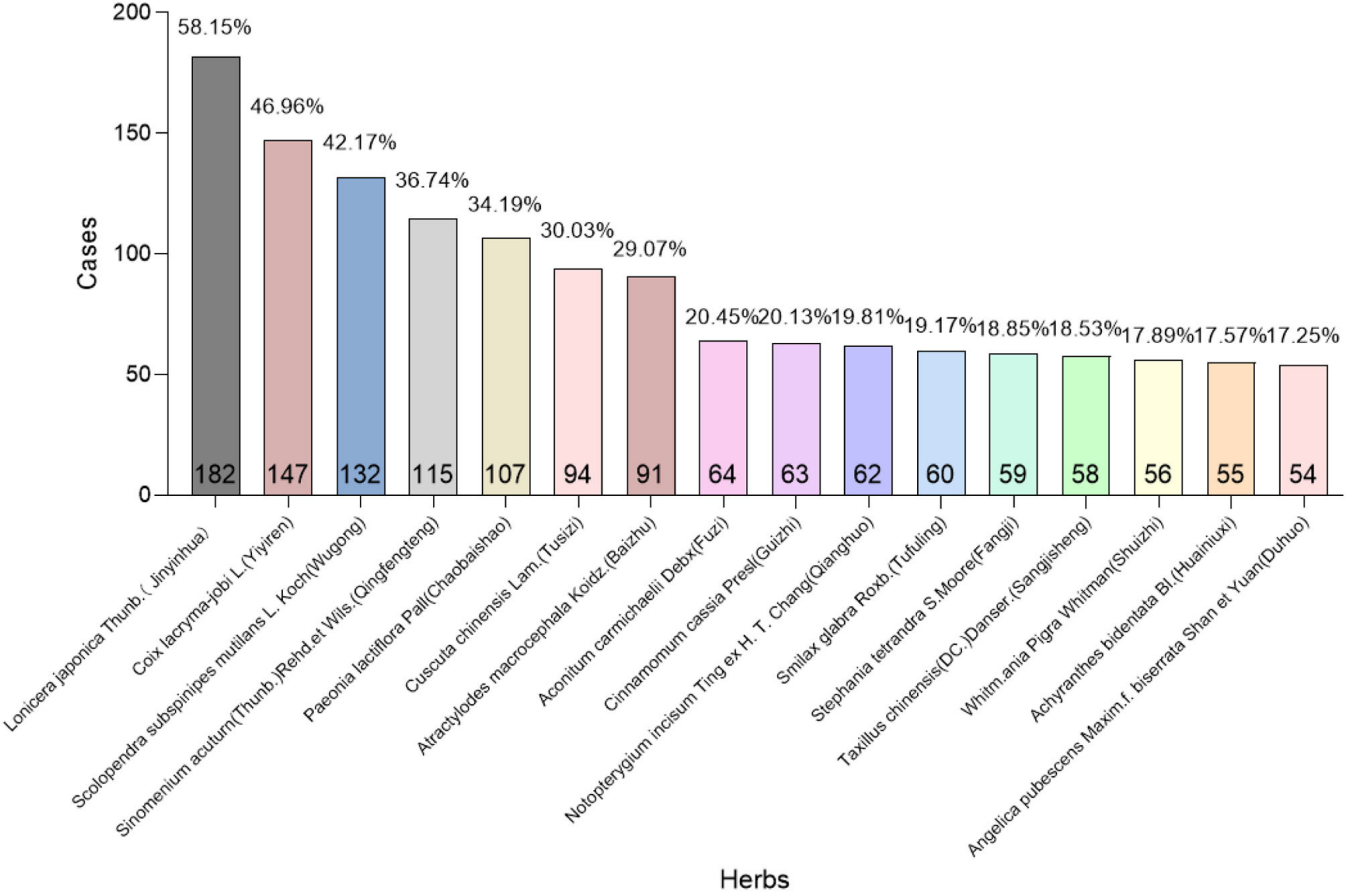
Screening of high frequency used herbs. *X*-axis: High frequency used herbs; *Y*-axis: Total times as well as its frequency that each herb appeared in 311 cases (At the bottom and top of the column, respectively). Herbs with their frequencies higher than 10% were enrolled in this study.

Different herbs were prescribed for different symptoms. High-frequency herbs may contribute to alleviating chronic pain symptoms. Then, we counted the times, as well as the frequency, of each herb prescribed in the records. Data show that high frequency used herbs are mainly consisted of “Jinyinhua” (182, 58.15%), “Yiyiren” (147, 46.96%), “Wugong” (132, 42.17%), “Qingfengteng” (115, 36.74%), “Chaobaishao” (107, 34.19%), “Tusizi” (94, 30.3%), “Baizhu” (91, 29.07%), “Fuzi” (64, 20.45%), “Guizhi” (63, 20.13%), “Qianghuo” (62, 19.81%), “Tufuling” (60, 19.17%), “Fangji” (59, 18.85%), “Sangjisheng” (58, 18.53%), “Shuizhi” (56, 17.89%), “Huainiuxi” (55, 17.57%), and “Duhuo” (54, 17.25%), respectively (Figure 4). We inferred that there might be some herbs with potential analgesic effects among these commonly used drugs.

### Classification and Analysis of High-Frequency Drugs

**Figure 5:**
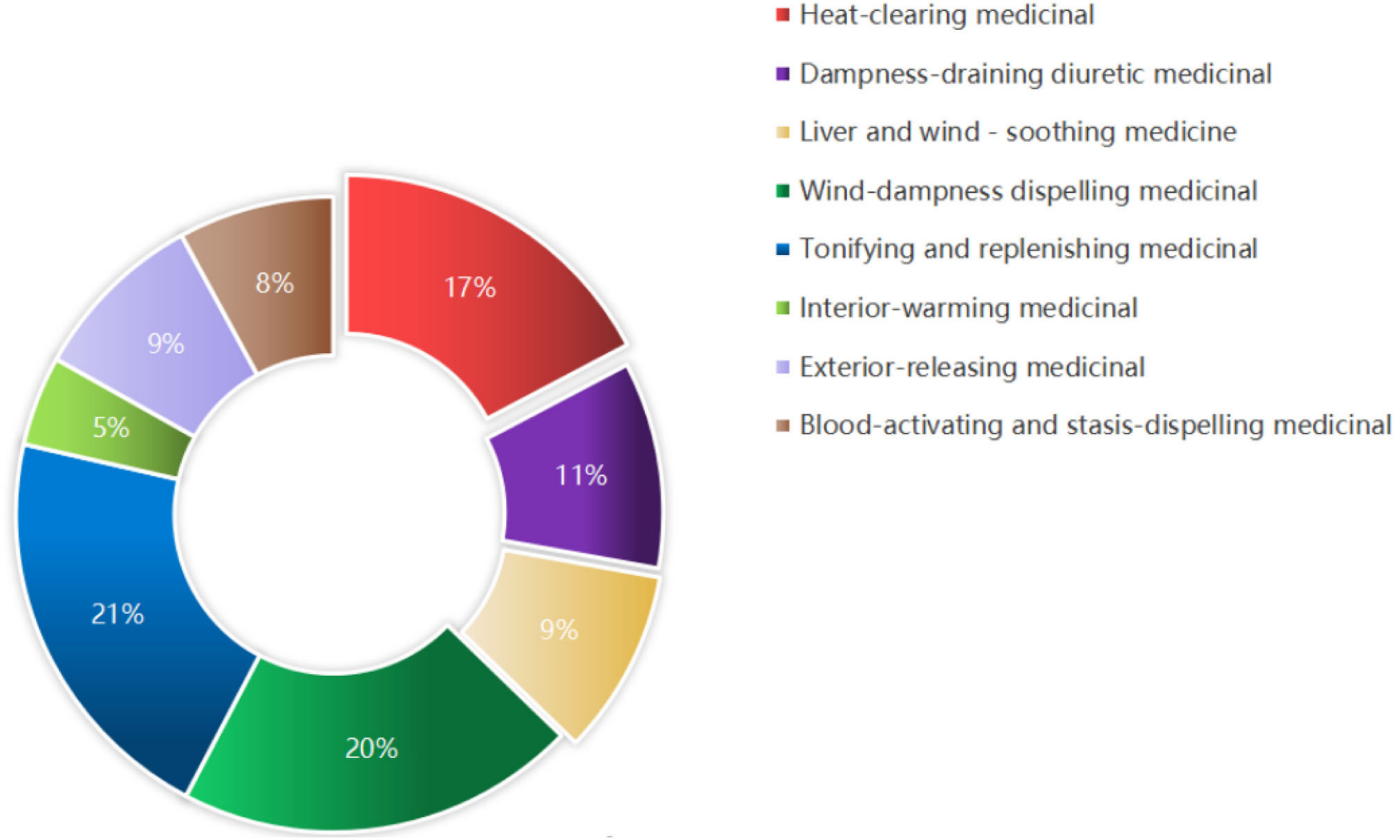
Different classifications of high frequency used herbs. Sixteen kinds of high frequency used herbs were classified into eight categories with their efficacy.

In Chinese traditional medications, the prescription of herbs was determined by the etiology and pathogenesis of chronic RA pain. To further explore the characteristics of these potential analgesics, combined with their efficacy, we found these high frequently used herbs belonging to eight categories. Then, we further calculated the proportion of each kind of category, respectively: “Heat-clearing medicinal” (17%), “Dampness-draining diuretic medicinal” (11%), “Liver and wind-soothing medicine” (9%), “Wind-dampness dispelling medicinal” (20%), “Tonifying and replenishing medicinal” (21%), “Interior-warming medicinal” (5%), “Exterior-releasing medicinal” (9%), and “Blood-activating and stasis-dispelling medicinal” (8%; Figure 5).

**Figure 6:**
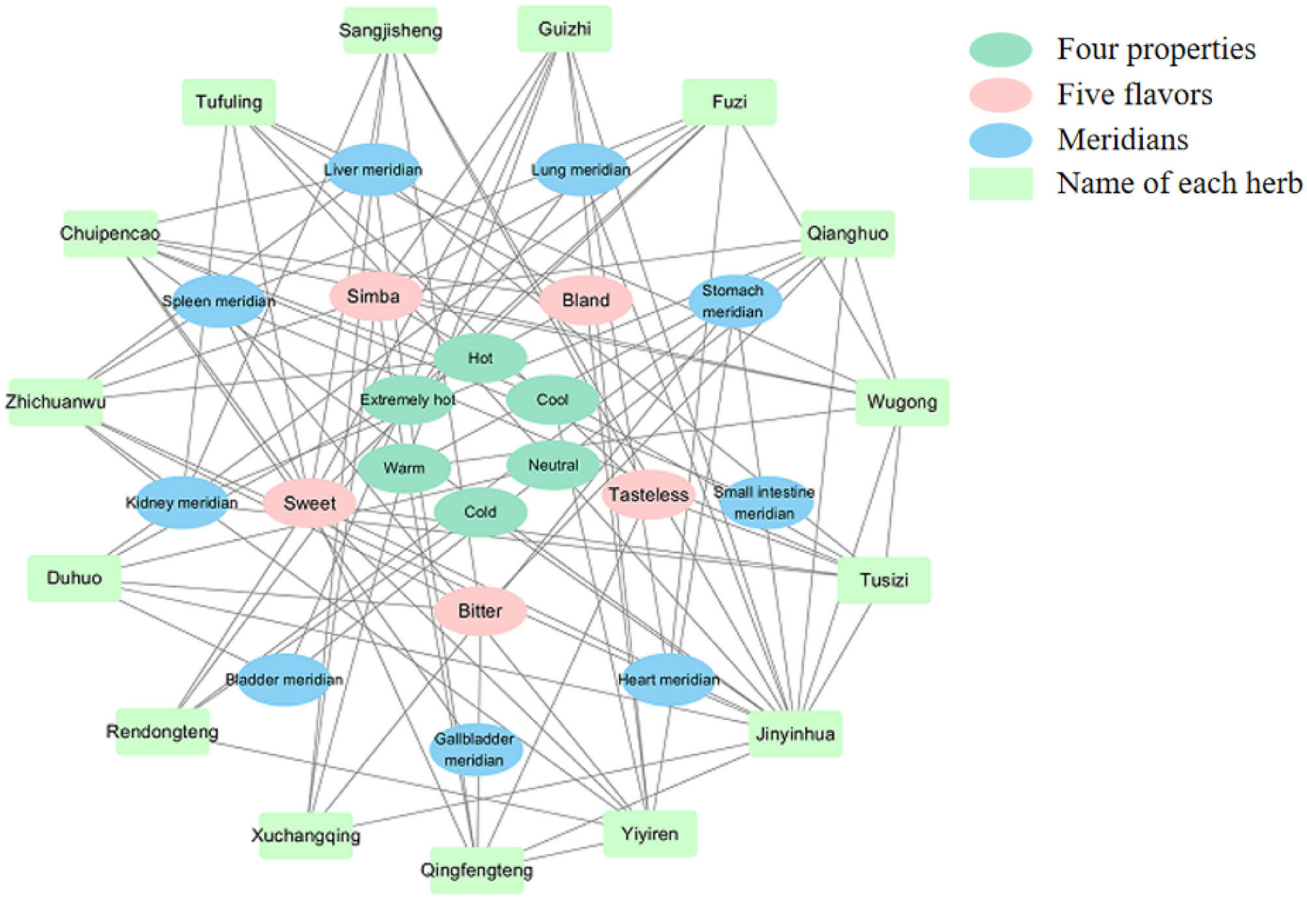
The network mapping of “Four properties,” “Five flavors,” and “Channel Tropism” of high-frequency herbs (bottle green: “Four properties”; pink: “Five flavors”; blue: “Meridians”; pale green: “Name of each herb”).

The “Four properties” (“Warm,” “Hot,” “Cool,” and “Cold”) and “Five flavors” (“Sour,” “Sweet,” “Bitter,” “Pungent,” and “Salty”), together with “Channel Tropism” (“Heart meridian,” “Liver meridian,” “Spleen meridian,” “Lung meridian,” “Kidney meridian,” “Small intestine meridian,” “Gallbladder meridian,” “Stomach meridian,” “large intestinal meridian,” “Bladder meridian,” and “Triple Energizer Meridian”), determine the function of herbs in Chinese traditional medication. To further study the characteristics of herbs prescribed in the records, according to The Pharmacopeia of the People's Republic of China (2020 edition), we classified all the herbs by “Four properties,” “Five flavors,” and “Channel Tropism.” We found that the flavors of “Sweet,” “Pungent,” “Bland,” “Tasteless,” and “Bitter” occupy a dominant position, while there are few herbs with four extreme properties like “Severe cold” or “Extremely hot,” suggesting that herbs with mild properties may benefit to chronic RA pain (Figure 6). Meanwhile, we found these herbs belonging to nine meridians, respectively: “Spleen meridian,” “Kidney meridian,” “Bladder meridian,” “Gallbladder meridian,” “Heart meridian,” “Small intestine meridian,” “Stomach meridian,” “Lung meridian,” and “Liver meridian” (Figure 6, Table 1), which indicated the targets that the herbs may affect.

**Table 1:** Main medicinal classification and frequency.

**Herbs**	**Frequency**	**Rate (%)**	**Property**	**Flavor**	**Meridian tropism**
*Lonicera japonica* Thunb.(Jinyinhua)	182	58.15	Cold	Sweet	Stomach, lung
*Coix lacryma-jobi* L .(Yiyiren)	147	46.96	Cool	Sweet, tasteless	Lung, spleen, stomach
*Scolopendra subspinipes* mutilans *L. Koch* (Wugong)	132	42.17	Warm	Pungent	Liver
*Sinomenium acuturn* (Thunb.)Rehd.et Wils.(Qingfengteng)	115	36.74	Neutral	Pungent, bitter	Liver, spleen
*Paeonia lactiflora* Pall(Chaobaishao)	107	34.19	Slightly cold	Bitter, sour	Liver, spleen
*Cuscuta chinensis* Lam.(Tusizi)	94	30.03	Neutral	Pungent, sweet	Liver, spleen, kidney
*Atractylodes macrocephala* Koidz.(Baizhu)	91	29.07	Warm	Bitter, sweet	Spleen, stomach
*Aconitum carmichaelii* Debx(Fuzi)	64	20.45	Extremely hot	Pungent, sweet	Heart, spleen, kidney
*Cinnamomum cassia* Presl(Guizhi)	63	20.13	Warm	Pungent, sweet	Lung, bladder, heart
*Notopterygium incisum* Ting ex H. T. Chang(Qianghuo)	62	19.81	Warm	Pungent, bitter	Bladder, kidney
*Smilax glabra* Roxb.(Tufuling)	60	19.17	Neutral	Sweet, tasteless	Liver, kidney
*Stephania tetrandra* S.Moore(Fangji)	59	18.85	Cold	Bitter	Bladder, lung
*Taxillus chinensis* (DC.)Danser.(Sangjisheng)	58	18.53	Neutral	Bitter, sweet	Liver, kidney
Whitm.ania Pigra Whitman(Shuizhi)	56	17.89	Neutral	Salty, bitter	Liver
*Achyranthes bidentata* Bl.(Huainiuxi)	55	17.57	Neutral	Bitter, sour, sweet	Liver, kidney
*Angelica pubescens* Maxim.f. *biserrata* Shan et Yuan(Duhuo)	54	17.25	Mild	Bitter, tasteless	Bladder, kidney

### Data Mining of Combination Patterns of Potential Analgesic Herbs Based on the Apriori Algorithm

**Figure 7:**
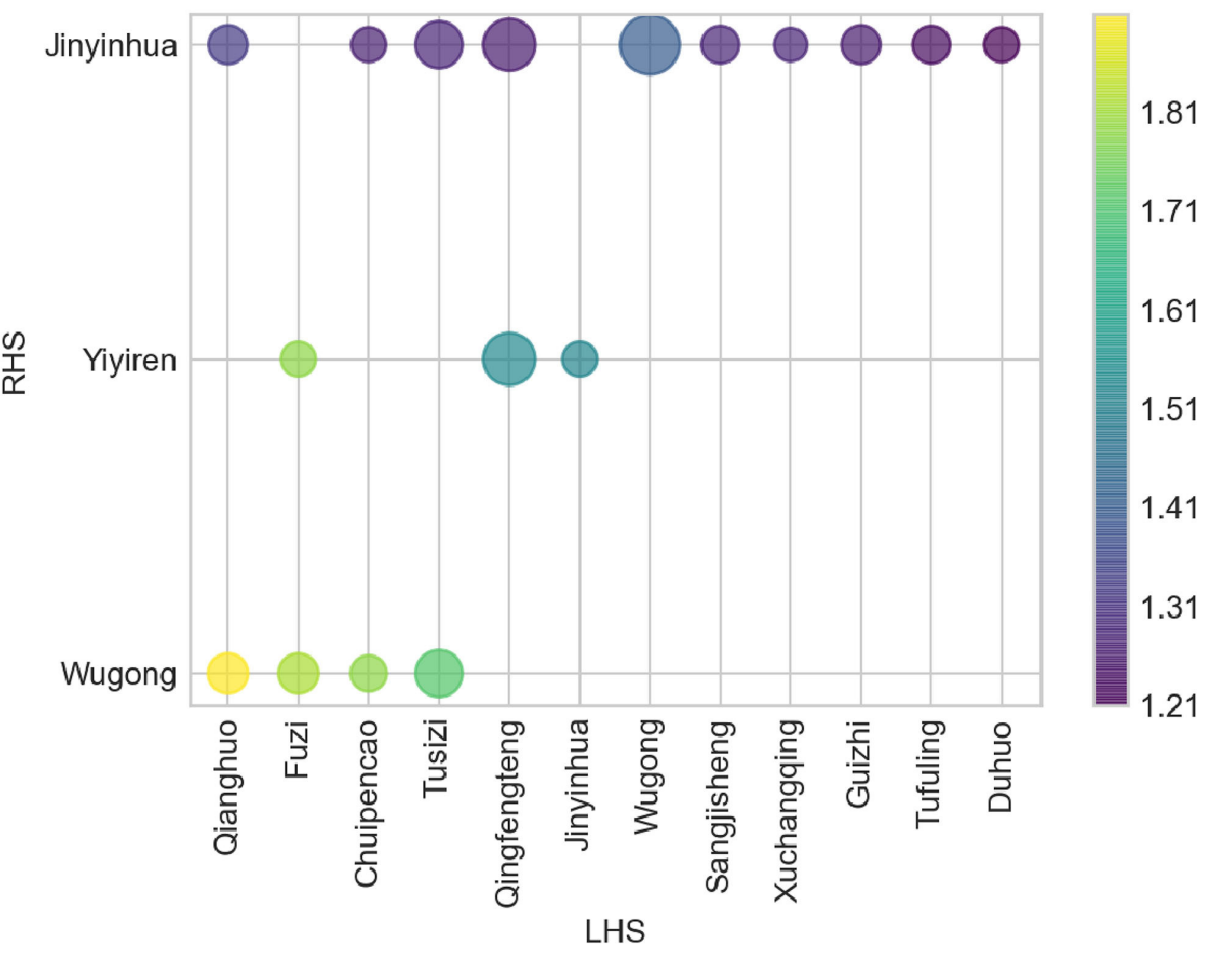
The bubble diagram of the grouping matrix for the 17 association rules based on the second-order association between different herbs. (*X*-axis: “LHS”; *Y*-axis: “RHS”; Size: “Support”; Color: “Lift.” Data were analyzed by using Apriori association rules and visualized by Python software).

The usage of combinations of potential analgesic herbs helps to improve drug efficacy. To further screen the potential compatibility relationship between different herbs, we used the second-order association of the Apriori Algorithm to conduct an association analysis on herbs (Figure 7, Supplementary Table 1). In this figure, the X-axis represents “LHS,” the Y-axis represents “RHS,” the size of the bubble represents the degree of the “Support,” and the color of the bubble represents the degree of “Lift.” The warmer the color of bubbles tends, the higher the correlation between “LHS” and “RHS” will be. Then, we found 17 association rules: (“Qianghuo,” “Wugong”), (“Fuzi,” “Wugong”), (“Chuipencao,” “Wugong”), (“Tusizi,” “Wugong”), (“Fuzi,” “Yiyiren”), (“Qingfengteng,” “Yiyiren”), (“Jinyinhua,” “Yiyiren”), (“Qianghuo,” “Jinyinhua”), (“Chuipencao,” “Jinyinhua”), (“Tusizi,” “Jinyinhua”), (“Qingfengteng,” “Jinyinhua”), (“Wugong,” “Jinyinhua”), (“Sangjisheng,” “Jinyinhua”), (“Xuchangqing,” “Jinyinhua”), (“Guizhi,” “Jinyinhua”), (“Tufuling,” “Jinyinhua”), and (“Duhuo,” “Jinyinhua”). We inferred that the coupled herbs with the strong correlation mentioned above would be more likely to have a synergistically therapeutic effect on pain symptoms.

**Figure 8:**
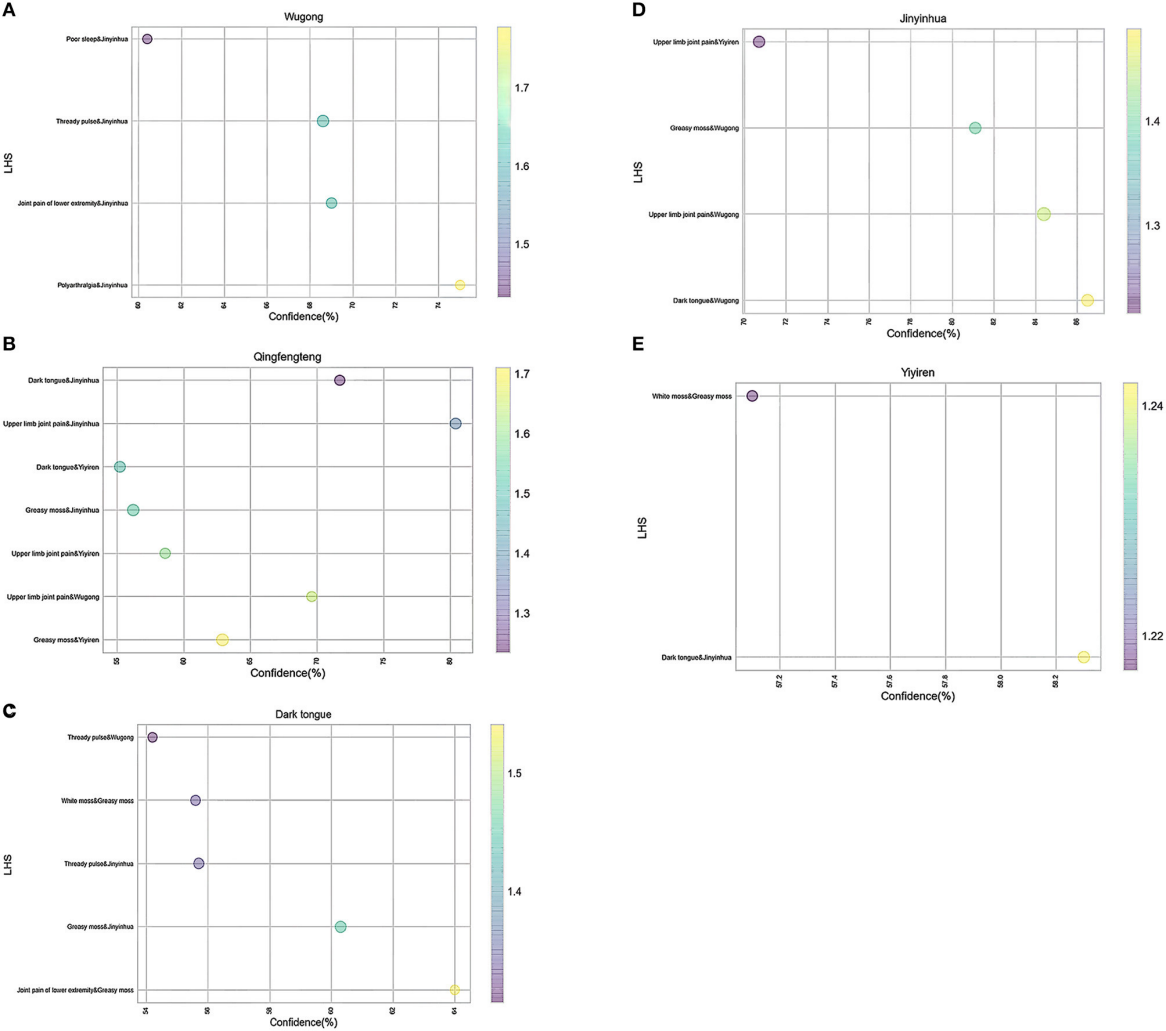
**(A–E)** The bubble diagram of the third-order association between herbs and symptoms. (Title: “RHS”; *X*-axis: “Confidence”; *Y*-axis: “LHS”; Size: “Support”; Color: “Lift.” Data were analyzed by using Apriori association rule method and visualized by Python software).

To further screen out the potential analgesic herbs, we performed a third-order association analysis of the herbs and symptoms mentioned above (Figure 8, Supplementary Table 4). Each title of the picture was regarded as “RHS,” while their corresponding herbs or symptoms were “LHS” (*Y*-axis). The *X*-axis represents the “Confidence” of associated medications and symptoms, while the size and color represent “Support” or “Lift,” respectively.

As shown in Figure 8, we divided the third-order correlation analysis graph into several parts. Corresponding to the previous research, it is effortless to find that “Polyarthralgia” and “Jinyinhua” were most correlated with “Wugong” (Figures 8A,D), indicating that the coupled herbs consisting of “Jinyinhua” and “Wugong” may have a therapeutic effect on the treatment of “Polyarthralgia.” Similarly, “Yiyiren” and “Qingfengteng” are potential coupled herbs used in “Greasy moss” (Figures 8B,E). These two herbs may help to attenuate the “Greasy moss” caused by “dampness.” Besides, the coupled symptoms “joint pain of lower extremities” and Greasy moss were correlated with “Dark tongue” (Figure 8C). Moreover, in all the item sets, the herbs “Jinyinhua,” “Wugong,” “Yiyiren,” and “Qingfengteng” appeared more frequently. These findings were consistent with our previous research (Supplementary Tables 2, 3). Taken together, herbs named “Jinyinhua,” “Yiyiren,” “Qingfengteng,” and “Wugong” respectively, seem to be helpful in attenuating “Polyarthralgia.”

### Recommendations for Combinations of Potential Analgesic Herbs Based on Traditional Cluster Analysis

To further substantiate this preliminary conclusion, we performed a system cluster analysis according to their occurrence in the records. Different color represents different frequencies, of the herbs, or symptoms that appeared in the records. The more frequently the herbs appeared in prescriptions, the more likely they were to be potential compatible drugs for the treatment of chronic pain in rheumatoid arthritis.

**Figure 9:**
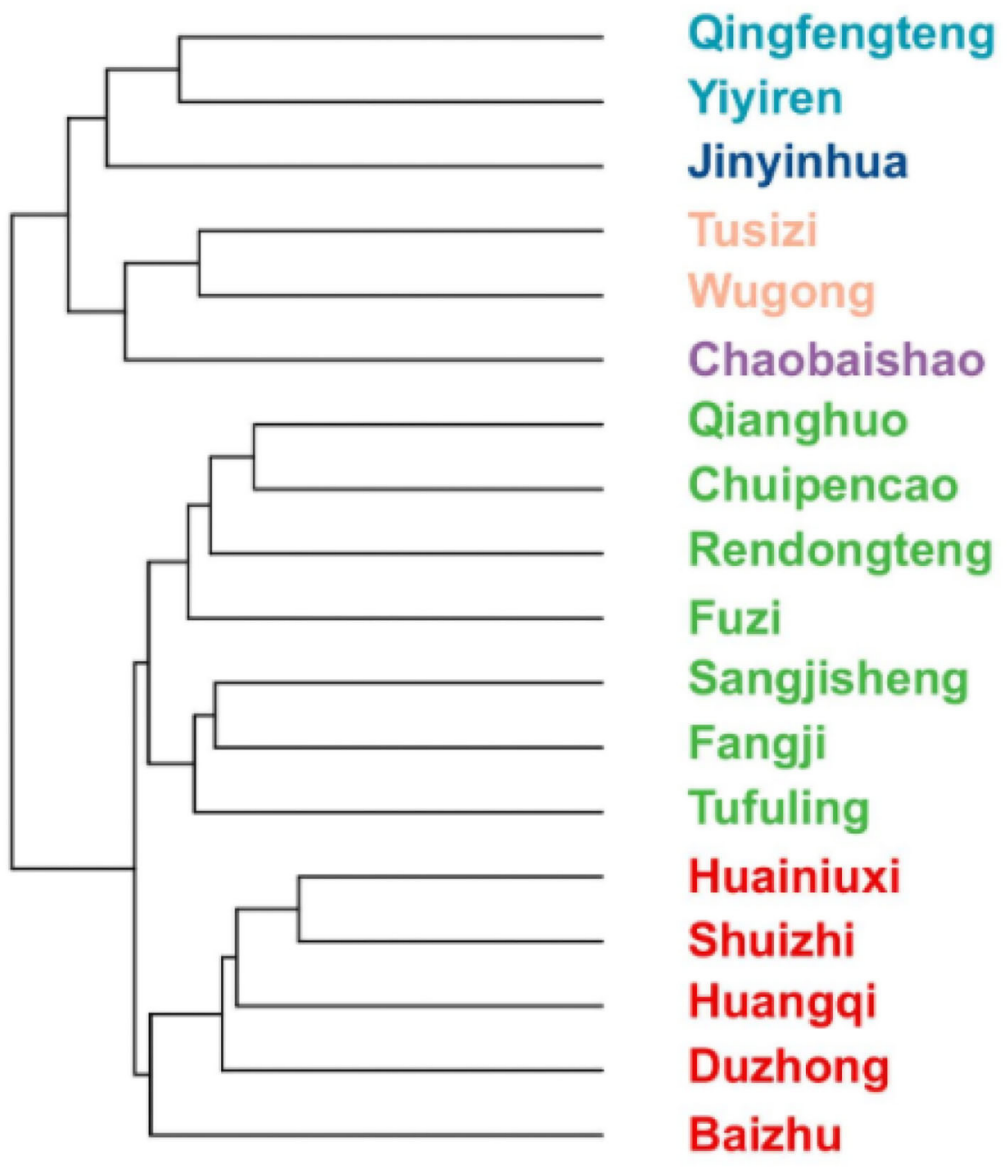
Tree diagram of systematic clustering of high frequency used drugs.

Just as Figure 9 shows, the herbs there are divided into three groups preliminarily: the first group is “Qingfengteng,” “Yiyiren,” “Jinyinhua,” “Tusizi,” “Wugong,” and “Chaobaishao.” The second group is “Qianghuo,” “Chuipencao,” “Rendongteng,” “Fuzi,” “Sangjisheng,” “Fangji,” and “Tufuling.” The last group is “Baizhu,” “Duzhong,” “Huangqi,” “Shuizhi,” and “Huainiuxi.”

Then, according to their color, we further divided the first group into several subgroups: “Qingfengteng” and “Yiyiren,” “Tusizi” and “Wugong”. Among them, “Jinyinhua” can be used with “Qingfengteng” and “Yiyiren,” while “Chaobaishao” can also combine with “Tusizi” and “Wugong.”

## Discussion

Chronic refractory pain of rheumatoid arthritis is difficult to control while using morphine to relieve persistent pain would lead to the side effects of addiction and drug resistance. Thus, finding alternative therapies and exploring potential analgesics is the key to treating pain in rheumatoid arthritis. In Traditional Chinese Medicine, research on the pain of rheumatoid arthritis has lasted for a long time. To further explore potential analgesics in TCM, Apriori association rule analysis was used in this study. In this study, first, we analyzed the main symptoms in the records of the 311 cases collected and found 16 kinds of frequently used herbs. Then, we screened out 17 association rules and four potential herbs (“Wugong,” “Jinyinhua,” “Qingfengteng,” and “Yiyiren”) with analgesic effects through the Apriori Algorithm. We further provided several recommendations for herbal combinations in the clinic by systematic clustering.

Through the analysis of 311 cases collected from “The Second Affiliated Hospital of Zhejiang Chinese Medical University,” we found “Polyarthralgia,” especially “Multi-articular pain,” is a prominent clinical symptom of chronic pain patients. The VAS scores of these patients decreased after TCM treatment, suggesting that analgesics were prescribed in their prescriptions. Besides, we screened out 16 kinds of different herbs prescribed with high frequencies: “Jinyinhua,” “Yiyiren,” “Wugong,” “Qingfengteng,” “Chaobaishao,” “Tusizi,” “Baizhu,” “Fuzi,” “Guizhi,” “Qianghuo,” “Tufuling,” “Fangji,” “Sangjisheng,” “Shuizhi,” “Huainiuxi,” and “Duhuo.” Combined with the pharmacopeia of the People's Republic of China (2020 edition), we classified these drugs according to their efficacy and found the ratio of “Heat-clearing medicinal,” “Dampness-draining diuretic medicine,” “Wind-dampness dispelling medicinal,” and “Tonifying and replenishing medicinal” are higher than 10%, suggesting that drugs with these efficiencies might play major roles in the treatment of chronic RA pain. Thus, we inferred that “Weak,” “Dampness,” “Wind,” and “Heat” are four potential pathogenic factors of chronic RA pain.

Meanwhile, we analyzed the characteristics of different herbs by studying the “Four properties,” “Five flavors,” and “Channel Tropism” of potential analgesic herbs (51). The flavors of herbs like “Sweet,” “Pungent,” “Bland,” “Tasteless,” and “Bitter” occupied a dominant position. In Chinese traditional medication, herbs with a “Sweet” taste usually have the effects of tonifying, while “Bitter-tasting medicine” usually has the effect of “Clearing heat.” Moreover, the herbs with “Pungent,” “Bland,” and “Tasteless” usually help to remove “Dampness” (52). These TCM conceptions are consistent with the results mentioned above in this study. Besides, we further found that a series of meridians like “Spleen meridian,” “Kidney meridian,” “Bladder meridian,” “Gallbladder meridian,” “Heart meridian,” “Small intestine meridian,” “Stomach meridian,” “Lung meridian,” and “Liver meridian” were promised to be the targets that these herbs may affect.

To further clarify the combination of herbs, we used the second-order correlation analysis based on the Apriori Algorithm analysis (47). Several potential coupled herbs were screened out. Although the color of bubbles shows that the data of “Lift” are all higher than “1,” they can still be classified into several levels of colors (Figure 8). The correlation between “Qianghuo” and “Wugong” is the highest, while “Qianghuo,” “Chuipencao,” “Tusizi,” “Qingfengteng,” “Wugong,” “Sangjisheng,” “Xuchangqing,” “Guizhi,” “Tufuling,” and “Duhuo” coupled with “Jinyinhua” belong to the fourth level. Besides, the third-order correlation analysis showed that “Polyarthralgia” and “Jinyinhua” were correlated with “Wugong,” indicating that these two herbs may be helpful for the treatment of “Polyarthralgia” (Figure 9). Patients with “Polyarthralgia,” especially the “Wandering Polyarthralgia,” are often thought to be attacked by “Wind evil” during the clinical treatment, referring to TCM conceptions (53). Correspondingly, “Clearing heat” and “Dispelling wind” might be the main principles of clinical treatment.

“Jinyinhua,” “Wugong,” “Yiyiren,” and “Qingfengteng” are the four frequently used herbs with their value ranks. Based on its anti-inflammatory properties, “Jinyinhua” can also exhibit analgesic efficacy *via* inhibiting NO, TNF-α, and IL-6 (54, 55).

Previous research showed that centipede peptides extracted from “Wugong,” identified as channel blockers of the voltage-gated sodium Nav1.7 and Cav2.2, can block pain signals *via* peripheral Transient receptor potential vanilloid 1^+^ (TRPV1^+^) neurons (56–61). However, Bianca found that mice treated with low doses of venom could induce mast cell degranulation and the secretion of MCP-1, IL-6, and IL-1β compared with PBS treated group, suggesting that some patients may be at risk of drug allergy after using “Wugong” (62). Although the coupled herbs consisting of “Jinyinhua” and “Wugong” may have a therapeutic effect on the treatment of “Polyarthralgia,” we speculated that “Jinyinhua” and “Wugong” were not the appropriate and versatile analgesic herbs.

As shown in Figure 8, we found that “Yiyiren” and “Qingfengteng” are potential coupled herbs used in “Greasy moss.” “Yiyiren,” a kind of useful “Dampness draining diuretic medicinal,” plays a key role in “Draining dampness” in TCM (63). According to pharmacological research, “the Coix seed oil,” extracted from “Yiyiren,” also has a powerful analgesic effect. Shinichi Tatsumi found that selective L5 spinal nerve transection mice treated with “Yiyiren” exerted an analgesic effect compared with untreated mice (64). Peirong Zhang found that patients with lung cancer injected with “the Coix seed oil” had increased tolerance to cancer pain (65). Different from “Yiyiren,” in addition to “Draining dampness,” “Qingfengteng” also has the role of “Dispelling wind” and “Clearing heat” in TCM. “Sinomenine,” the active ingredient of “Qingfengteng” has a powerful central analgesic effect without the side effects of drug addiction (66, 67). “Sinomenine” can bilaterally regulate the Treg cells and Th17 cells and shows anti-inflammatory and analgesic effects in the synovial site of the rat joints. Huang Feng's group also reported that “Sinomenine” regulates both Th1 cells and Th2 cells and acts through its analgesic role *via* NF-κB (68). Moreover, it reported that “Sinomenine,” acts as a ligand and agonist of AhR, can potentiate Treg activity, suggesting that the immunomodulatory effect and anti-arthritic effect of “Sinomenine” manifested in an AhR-dependent manner (69, 70). Besides, “Sinomenine” can inhibit 12 kinds of inflammatory factors released by macrophages and plays a peripheral anti-inflammatory role (71). Meanwhile, by inhibiting the release of inflammatory factors, it can reduce the combination of nociceptive substances and peripheral nociceptive receptors (72–74). Thus, in the peripheral, “Sinomenine” can play an analgesic role indirectly (71).

Taken together, “Weakness,” “Wind,” “Dampness,” and “Heat” are four major pathogenic factors of chronic pain consistent with the results mentioned above. “Qingfengteng,” as well as its active ingredient—“Sinomenine,” can alleviate chronic pain through anti-inflammatory and non-anti-inflammatory pathways. “Qingfengteng” seems to be more helpful in attenuating chronic RA pain and is promised to be an essential drug for treating chronic pain of rheumatoid arthritis.

The combination of the herbs analyzed in Figure 9 gives novel guidance to the clinic. When the “Wind evil” dominates, “Qingfengteng” and “Wugong” may become the best choice. When the “Heat evil” occupies, “Qingfengteng” and “Jinyinhua” can exert their effect of “Clearing heat.” When the “Dampness” is given priority, “Qingfengteng,” “Yiyiren,” and “Chaobaishao” are considered for prescription. When patients feel “Weak,” “Qingfengteng” combined with “Tusizi” and “Chaobaishao” seems more reasonable.

This study had screened out the potential compatibility combination patterns of drugs and corresponding analgesics for the treatment of rheumatoid arthritis, but the study leaves much room for further improvement. Due to the cost and conditions of clinical trials, RCT research cannot be achieved. The system “Rheumatism Intelligent Auxiliary diagnosis and treatment system” is developed by our platform, which is not free for global scholars. TCM is just a little part of the world of ethnic medicine, and we believe that there exist other treatments for the pain of rheumatoid arthritis.

## Conclusions

In this study, we collected 311 cases and screened out 16 kinds of high frequency used drugs from the prescriptions. The pain symptoms may be induced by “Weakness,” “Wind,” “Dampness,” and “Heat.” Four potential analgesic herbs (“Wugong,” “Qingfengteng,” “Jinyinhua,” and “Yiyiren”) with their combinations may help to alleviate RA pain with moderate activity.

## Data Availability Statement

The raw data supporting the conclusions of this article will be made available by the authors, without undue reservation.

## Ethics Statement

The studies involving human participants were reviewed and approved by Ethics Committee of Zhejiang Chinese Medicine University Zhejiang Chinese Medicine University. The patients/participants provided their written informed consent to participate in this study. Written informed consent was obtained from the individual(s) for the publication of any potentially identifiable images or data included in this article.

## Author Contributions

W-dL: conceptualization, methodology, data curation, visualization, and writing—original draft. D-mL, SW, J-jW, and S-jC: software. JY: validation, writing—review and editing, and funding acquisition. LH: software and validation. M-zZ: software and formal analysis. Y-pJ: visualization. C-pW: validation, funding acquisition, and supervision. YJ: supervision.

## Funding

This study was supported by the National key Research and Development Program (NO.2018YFC1705500), the National Natural Science Foundation (NO.81971052), and the Zhejiang Provincial Natural Science Foundation of China (LY22H280008).

## Conflict of Interest

The authors declare that the research was conducted in the absence of any commercial or financial relationships that could be construed as a potential conflict of interest.

## Publisher's Note

All claims expressed in this article are solely those of the authors and do not necessarily represent those of their affiliated organizations, or those of the publisher, the editors and the reviewers. Any product that may be evaluated in this article, or claim that may be made by its manufacturer, is not guaranteed or endorsed by the publisher.

## Supplementary Material

Supplementary Table 1 The second-order association analysis of high frequency used herbs (Confidence >50, Support >10, Lift>1.2).

Supplementary Table 2 The third-order association analysis of high frequency used herbs (Confidence >50, Support >10, Lift>1.2).

Supplementary Table 3 The second-order association analysis between symptoms and high frequency used herbs (Confidence >50, Support >10, Lift>1.2).

Supplementary Table 4 The third-order association analysis between symptoms and high frequency used herbs (Confidence >50, Support >10, Lift>1.2).

Supplementary Figure 1 Changes in blood tests before and after TCM treatment. **(a–d)**: The Changes of ESR **(a)**, hsCRP **(b)**, RF **(c)**, and Anti-CCP Antibody **(d)** before and after TCM treatment, respectively.
