# Supplementary material for: An Apriori Algorithm-Based Association Analysis of Analgesic Drugs in Chinese Medicine Prescriptions Recorded From Patients With Rheumatoid Arthritis Pain
Source: Front Pain Res (Lausanne). 2022 Jul 25;3:937259. doi: 10.3389/fpain.2022.937259 (PMC9358686; doi:10.3389/fpain.2022.937259)
Supplement: Supplementary Table 1 — The second-order association analysis of high frequency used herbs (Confidence >50, Support >10, Lift>1.2). [file Data_Sheet_1.docx]

Supplementary Table 1: The second order association analysis of high frequency used herbs. (Confidence > 50, Support >10, Lift>1.2)

| The second order association analysis of high frequency used herbs | | | | |
| --- | --- | --- | --- | --- |
| LHS | RHS | Confidence | Support | Lift |
| Qianghuo | Wugong | 81 | 16 | 1.91 |
| Fuzi | Wugong | 77 | 16 | 1.82 |
| Chuipencao | Wugong | 75 | 13 | 1.79 |
| Zhichuanwu | Yiyiren | 84 | 12 | 1.79 |
| Tusizi | Wugong | 72 | 22 | 1.72 |
| Qingfengteng | Yiyiren | 72 | 27 | 1.54 |
| Rendongteng | Yiyiren | 72 | 12 | 1.53 |
| Wugong | Jinyinhua | 82 | 35 | 1.41 |
| Qianghuo | Jinyinhua | 77 | 15 | 1.33 |
| Tusizi | Jinyinhua | 74 | 22 | 1.28 |
| Sangjisheng | Jinyinhua | 74 | 14 | 1.28 |
| Xuchangqing | Jinyinhua | 74 | 11 | 1.28 |
| Chuipencao | Jinyinhua | 74 | 12 | 1.27 |
| Qingfengteng | Jinyinhua | 73 | 27 | 1.26 |
| Guizhi | Jinyinhua | 73 | 15 | 1.26 |
| Tufuling | Jinyinhua | 72 | 14 | 1.23 |
| Duhuo | Jinyinhua | 71 | 12 | 1.21 |

Supplementary Table 2: The third order association analysis of high frequency used herbs. (Confidence > 50, Support >10, Lift>1.2)

| The third order association analysis of high frequency used herbs | | | | |
| --- | --- | --- | --- | --- |
| LHS | RHS | Confidence | Support | Lift |
| Chaobaishao&Tusizi | Wugong | 92 | 11 | 2.18 |
| Fuzi&Jinyinhua | Wugong | 91 | 12 | 2.15 |
| Guizhi&Jinyinhua | Wugong | 87 | 13 | 2.06 |
| Wugong&Qingfengteng | Jinyinhua | 88 | 16 | 1.5 |
| Tusizi&Wugong | Jinyinhua | 82 | 18 | 1.42 |
| Yiyiren&Wugong | Jinyinhua | 83 | 17 | 1.42 |
| Chaobaishao&Wugong | Jinyinhua | 77 | 15 | 1.33 |
| Qianghuo&Wugong | Jinyinhua | 76 | 12 | 1.31 |

Supplementary Table 3: The second order association analysis between symptoms and high frequency used herbs. (Confidence > 50, Support >10, Lift>1.2)

| The second order association analysis between symptoms and high frequency used herbs | | | | |
| --- | --- | --- | --- | --- |
| LHS | RHS | Confidence | Support | Lift |
| Lumbosacral pain | Shuizhi | 50 | 10.2 | 2.795 |
| Morning paralysis | Wugong | 70.2 | 10.5 | 1.665 |
| Qianghuo | Upper limb joint pain | 51.6 | 10.2 | 1.599 |
| Upper limb joint pain | Wugong | 63.4 | 20.4 | 1.503 |
| Polyarthralgia | Wugong | 61.2 | 13.1 | 1.451 |
| Inflexible body movement | Qingfengteng | 53 | 11.2 | 1.443 |
| Morning paralysis | Jinyinhua | 80.9 | 12.1 | 1.39 |
| Upper limb joint pain | Jinyinhua | 72.3 | 23.3 | 1.243 |
| Lumbosacral pain | Yiyiren | 57.8 | 11.8 | 1.231 |
| Inflexible body movement | Yiyiren | 57.6 | 12.1 | 1.226 |
| Upper limb joint pain | Yiyiren | 57.4 | 18.5 | 1.223 |
| Thready pulse | Wugong | 50.9 | 18.8 | 1.206 |

Supplementary Table 4: The third order association analysis between symptoms and high frequency used herbs. (Confidence > 50, Support >10, Lift>1.2)

| The third order association analysis between symptoms and high frequency used herbs | | | | |
| --- | --- | --- | --- | --- |
| LHS | RHS | Confidence | Support | Lift |
| Polyarthralgia&Jinyinhua | Wugong | 75 | 10.5 | 1.78 |
| Greasy moss&Yiyiren | Qingfengteng | 62.9 | 14.1 | 1.71 |
| Upper limb joint pain&Wugong | Qingfengteng | 69.6 | 10.2 | 1.65 |
| Joint pain of lower extremity&Jinyinhua | Wugong | 69 | 12.8 | 1.64 |
| Thready pulse&Jinyinhua | Wugong | 68.6 | 15.3 | 1.63 |
| Upper limb joint pain&Yiyiren | Qingfengteng | 58.6 | 10.9 | 1.60 |
| Joint pain of lower extremity&Greasy moss | Dark tongue | 64 | 10.2 | 1.54 |
| Greasy moss&Jinyinhua | Qingfengteng | 56.2 | 13.1 | 1.53 |
| Dark tongue&Yiyiren | Qingfengteng | 55.2 | 11.8 | 1.50 |
| Dark tongue&Wugong | Jinyinhua | 86.5 | 14.4 | 1.49 |
| Greasy moss&Jinyinhua | Dark tongue | 60.3 | 14.1 | 1.45 |
| Upper limb joint pain&Wugong | Jinyinhua | 84.4 | 17.3 | 1.45 |
| Poor sleep&Jinyinhua | Wugong | 60.4 | 10.2 | 1.43 |
| Greasy moss&Wugong | Jinyinhua | 81.1 | 13.7 | 1.40 |
| Upper limb joint pain&Jinyinhua | Qingfengteng | 80.4 | 11.8 | 1.38 |
| Thready pulse&Jinyinhua | Dark tongue | 55.7 | 12.5 | 1.34 |
| White moss&Greasy moss | Dark tongue | 55.6 | 11.2 | 1.34 |
| Thready pulse&Wugong | Dark tongue | 54.2 | 10.2 | 1.31 |
| Dark tongue&Jinyinhua | Yiyiren | 58.3 | 13.4 | 1.24 |
| Dark tongue&Jinyinhua | Qingfengteng | 71.7 | 10.5 | 1.23 |
| White moss&Greasy moss | Yiyiren | 57.1 | 11.5 | 1.22 |
| Upper limb joint pain&Yiyiren | Jinyinhua | 70.7 | 13.1 | 1.22 |
