# Supplementary figures and images for: An Apriori Algorithm-Based Association Analysis of Analgesic Drugs in Chinese Medicine Prescriptions Recorded From Patients With Rheumatoid Arthritis Pain
Source: Front Pain Res (Lausanne). 2022 Jul 25;3:937259. doi: 10.3389/fpain.2022.937259 (PMC9358686; doi:10.3389/fpain.2022.937259)

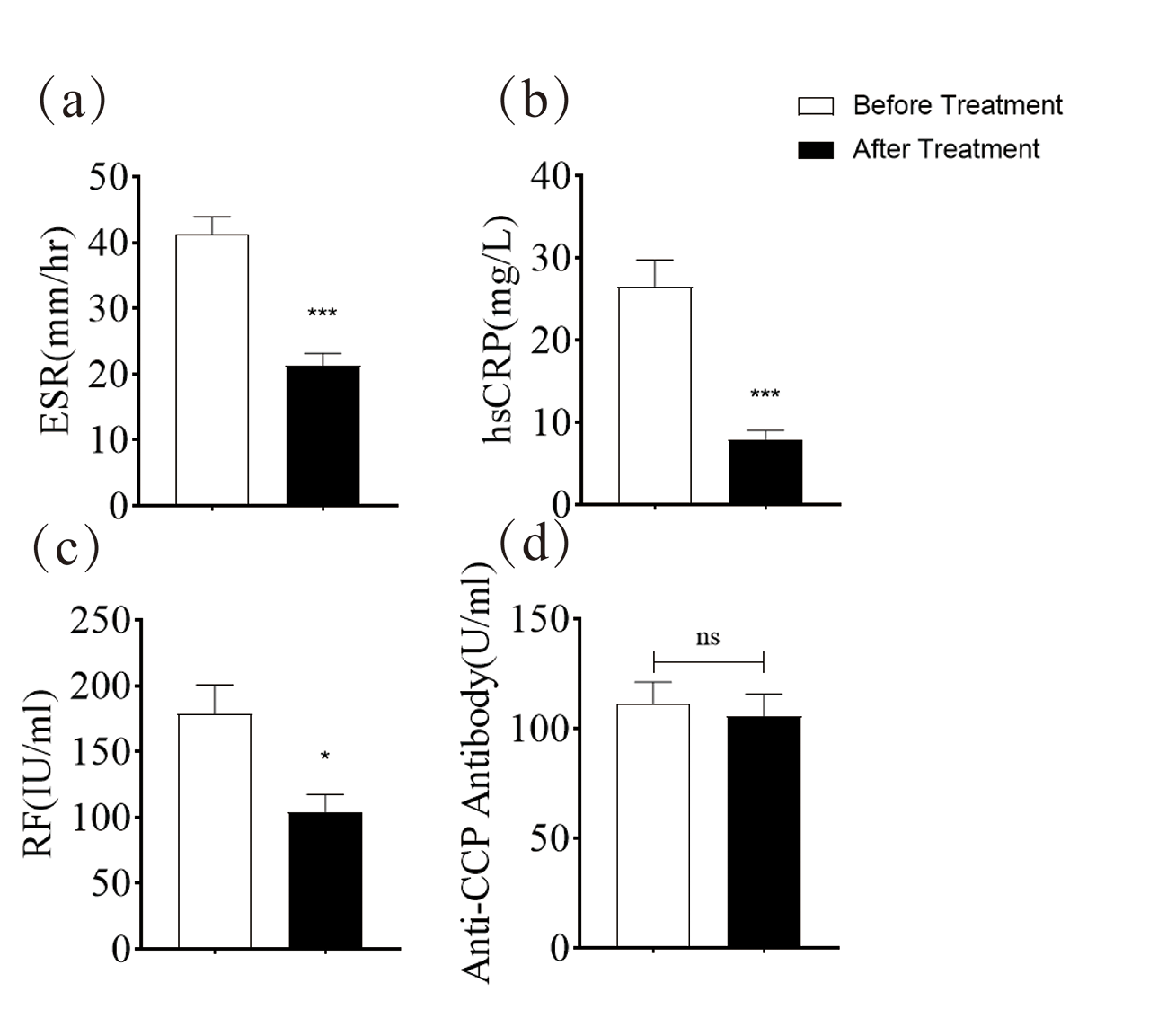

Supplement: Supplementary Figure 1 — Changes in blood tests before and after TCM treatment. (a–d): The Changes of ESR (a), hsCRP (b), RF (c), and Anti-CCP Antibody (d) before and after TCM treatment, respectively. [file Image_1.tif]
